# Childhood Trauma and Insomnia Increase Suicidal Ideation in Schizophrenia Patients: A Cross-Sectional Study

**DOI:** 10.3389/fpsyt.2021.769743

**Published:** 2021-11-11

**Authors:** Yaoyao Zhang, Xinyu Fang, Bei Tang, Kaili Fan, Na Wen, Ke Zhao, Weiqian Xu, Wei Tang, Yi Chen

**Affiliations:** ^1^Department of Psychiatry, Wenzhou Medical University, Wenzhou, China; ^2^Department of Psychiatry, Affiliated Nanjing Brain Hospital, Nanjing Medical University, Nanjing, China; ^3^Children's Hospital of Nanjing Medical University, Nanjing, China; ^4^Department of Psychiatry, The Affiliated Kangning Hospital of Wenzhou Medical University, Wenzhou, China; ^5^Department of Psychiatry, The Second People's Hospital of Taizhou, Taizhou, China

**Keywords:** suicide, childhood maltreatment, schizophrenia, insomnia, physical neglect

## Abstract

**Objectives:** This study aimed to investigate the effect of childhood trauma, especially its specific dimensions, and clinical risk factors for suicidal ideation in patients with schizophrenia.

**Methods:** A total of 83 inpatients with schizophrenia were enrolled and divided into two groups: with suicidal ideation (*n* = 33) and without suicidal ideation (*n* = 50). All participants were administered the Childhood Trauma Questionnaire-Short Form, the Insomnia Severity Index, the Beck Scale for Suicide Ideation, the Modified Overt Aggression Scales, the auditory hallucination rating scale, the Hamilton Rating Scale of Depression and the Positive and Negative Syndrome Scale.

**Results:** In our sample, 39.8% of the subjects had suicidal ideation, and 60.6% of them had suffered from childhood trauma. Patients with suicidal ideation had a higher Insomnia Severity Index score, Physical neglect score, the Childhood Trauma Questionnaire-Short Form total score (all *P* < 0.05) compared to those without. The logistic regression analysis revealed that physical neglect in Childhood Trauma Questionnaire was significantly associated with suicidal ideation (OR = 5.46, *P* < 0.05, 95% CI = 0.007–0.483). Further stepwise multiple linear regression identified that insomnia (β = 0.272, *P* = 0.011) and physical neglect (β = 0.257, *P* = 0.017) were strong risk factors for the severity of suicidal ideation in patients with schizophrenia. Mediation analysis showed that insomnia played a complete mediating role between physical neglect and suicidal ideation.

**Conclusion:** Our results indicate that childhood maltreatment of physical neglect is a strong independent risk factor for suicidal ideation in schizophrenia. The risk is probably aggravated by the poor quality of sleep. Early screening and psychosocial treatment are recommended for psychotic individuals with a trauma history.

## Introduction

Schizophrenia is a serious psychiatric disorder of unknown etiology and is associated with high mortality and morbidity (1–3). Suicide is one of the major causes of death among individuals with schizophrenia (4). About 5.6% of patients with schizophrenia die by suicide, and about 10–50% have suicidal ideation or attempt (5, 6). With high mortality rates, the adverse effects on the family and society of this pathology are significant. Therefore, previous studies were actively looking for clinical and psychobiological risk factors for suicidal behavior, thereby reducing the occurrence of suicides and implementing suicide intervention early.

Early researches have documented the association between suicide risk and demographic characteristics and clinical features in schizophrenia, including severe depressive symptoms (7), great cognitive ability (8), male gender, young age, higher intelligence quotient and family history of suicide attempts (9), alcohol and substance use disorder, smoking, among others (4, 6), but there is an abundance of research findings that failed to confirm those associations (10). Active hallucinations, as a risk factor for suicide, also had a strong evidential basis (11, 12). In addition to the demographic and clinical associations, rising evidence has supported an important role of childhood trauma (CT) and insomnia in predicting suicide risk in schizophrenia.

It has been found that those who suffered from CT have a susceptibility factor for developing schizophrenia compared to children without CT (13, 14). Interestingly, the suicidal risk was stronger among individuals with depression who reported a history of CT (15). However, previous studies on the relationship between CT and suicide in schizophrenia have been inconsistent. Some research shows that CT has nothing to do with attempted suicide in schizophrenia (16), but other studies have found that CT is a strong predictor of suicide in schizophrenia (17–19). Further studies find that suicide attempts are associated with all CT dimensions, such as physical, sexual, psychological abuse, and neglect, except for physical neglect (20), while other studies find that physical neglect and sexual abuse are indeed associated (21, 22). Much of the literature on suicidality in patients with schizophrenia considers CT but does not specify which aspect, or only considers one aspect of CT. Therefore, it is necessary to explore the relationship between CT and suicide in schizophrenia, at the same time, to further explore what aspects of CT are more related to suicide risk.

Moreover, insomnia is also commonly seen in schizophrenia patients, but it is easily ignored in those patients (23). Numerous studies reported that insomnia, as a core symptom of depression, has been associated with an increased risk of suicidal ideation (24, 25). This relationship is also found in patients with anxiety and post-traumatic stress disorder (26). Although insomnia is closely related to suicide in other mental illnesses, there is a paucity of data on this association in patients with schizophrenia. Hence, more research is needed to understand the association between insomnia and suicide risk in schizophrenia patients.

As noted in a review by Kajeepeta et al. there is extensive evidence that the impact of CT on sleep can last into adulthood (27). A study comprised of 9,582 samples observed that the risk of insomnia differed based on the age at first exposure to adversity as well as the type of adversity (28). Moreover, a cross-sectional questionnaire survey also provided that impaired subjective sleep was significantly predicted by child maltreatment experiences (29).

In the present study, we aimed to explore the relationship between CT, clinical factors such as insomnia, and suicide ideation in schizophrenia patients, and further to identify which aspects of the trauma are most associated with suicidal ideation in these patients. Based on the available literature, we hypothesized that patients with a history of CT have stronger suicidal ideation and that the relationship between CT and suicidal ideation is mediated by impaired sleep quality.

## Methods

### Participants

A total of 83 inpatients with schizophrenia at the Affiliated Kangning Hospital of Wenzhou Medical University. were involved in our study between December 2018 and December 2019. All patients met the following criteria: (1) a diagnosis of schizophrenia according to the Diagnostic and Statistical Manual of Mental Disorders, Fourth Edition (DSM-IV) (30); (2) age 18–75 years old; and (3) Han Chinese ethnicity. Patients were excluded if they had: (1) severe cardiovascular, hepatic, or renal diseases; (2) pregnancy or breastfeeding; and (3) an intellectual disability. This study was performed in strict accordance with the Declaration of Helsinki and all other relevant national and international regulations. The study protocol was approved by the Medical Ethics Committee of the Affiliated Kangning Hospital of Wenzhou Medical University. All participants signed informed consent before the formal study. Based on their scores of items 4 or 5 on the Beck Scale for Suicide Ideation, we divided all patients with schizophrenia into schizophrenia with suicidal ideation group and schizophrenia without suicidal ideation group. A score of item 4 or 5 > 1 indicates patients with suicidal ideation, and only when the scores of item 4 or 5 were 1, patients were considered as having no suicidal ideation.

### Measures

Data on demographic characteristics, physical disease, and history of tobacco and alcohol were collected utilizing validated, self-administered questionnaires. Body mass index was calculated as weight in kg/square of height in meters.

We used the Childhood Trauma Questionnaire-Short Form (CTQ-SF) (31) to evaluate the occurrence of childhood maltreatment and its severity in schizophrenia patients. The CTQ-SF contains 28 items to assess CT dimensions including sexual abuse (SA), physical abuse (PA), emotional abuse (EA), physical neglect (PN), and emotional neglect (EN), which is a widely used and validated retrospective self-reported to assess childhood trauma exposures. Total sum scores can range between 25 and 125 points. The higher the score, the more frequent and therefore severe maltreatment events were experienced. For individual CT dimensions, a score ≥ 10 denotes patients with high levels of physical abuse and physical neglect, and a score ≥ 8 denotes patients with high levels of sexual abuse; A score ≥ 13 indicates patients with high levels of emotional abuse, and a score ≥ 15 indicates patients with high levels of emotional neglect.

The Insomnia Severity Index (ISI), which has good internal consistency, captures the severity and impact of insomnia symptoms (32). The screening measure for insomnia consists of seven items that are rated on a five-point rating scale from 0 to 4, resulting in a sum score between 0 and 28. The higher score of ISI indicates the worse of sleep quality, and a score of 7 and more in ISI was categorized as insomnia.

The Beck Scale for Suicide Ideation (BSSI) was used to assess the severity of suicidal ideation in the patients. This scale consists of 19 items designed to be self-scored on a 1–3 point scale, the average of the first 5 items assesses the intensity of suicidal ideation, and the next items are used to assess the risk of suicide, with higher scores indicating higher levels of suicidal ideation and risk. The reliability and validity of the BSSI have been confirmed to be appropriate (33).

In addition, The Positive and Negative Syndrome Scale (PANSS) (34), the Hamilton Rating Scale of Depression (HAMD) (35), the Modified Overt Aggression Scales (MOAS) (36) and, the auditory hallucination rating scale (AHRS) (37) were all used to evaluate the clinical symptoms including positive, negative symptoms, especially the auditory hallucination, as well as aggressive behavior and depressive symptoms.

All these scales have been widely used to assess clinical symptoms in patients with schizophrenia. Clinical evaluations were carried out by experienced psychiatrists who were well-trained for this project, and repeated assessments revealed that a correlation coefficient > 0.8 was maintained.

### Statistical Analyses

Statistical analyses were performed using SPSS software version 26.0 (SPSS, 146 Chicago, IL). First, we used the Kolmogorov–Smirnov one-sample test to test for the normality of the variables. Second, independent samples *t*-tests and Pearson's chi-square test were performed to compare the differences between patients with and without suicidal ideation (SI) in terms of sociodemographic and clinical characteristics. If the results of the Student's *t*-test were significant, we then performed the analysis of covariance (ANCOVA) to control for potential confounding factors, such as sex, education level and smoking history. We further used SI as the dependent variable to perform a stepwise logistic regression analysis with the “forward: Conditional” method to identify factors independently associated with SI in patients with schizophrenia, with the categorical variables that were statistically significant between groups (physical neglect and insomnia) as independent variables, and age, sex, educational status and smoking history as covariates. Then, a stepwise linear regression analysis (forward) was performed to explore the risk factors for the severity of suicidal ideation (dependent variable) in schizophrenia patients, with variables that were statistically significant between groups (physical neglect and insomnia severity) as independent variables, and age, sex, education status and smoking history were also controlled. Finally, A plug-in program process was also applied in SPSS to perform a significant mediation effect test and set up bootstrap sampling for bias-corrected 5,000 times. Standardized coefficients and 95% confidence intervals (95% CI) were calculated. All tests were two-tailed, and a value of *p* < 0.05 was considered significant.

## Results

Table 1 presents the demographic characteristics of schizophrenic patients with or without suicidal ideation. Among 83 patients with schizophrenia, 33 (39.8%) with SI and 50 (60.2%) without SI. There were significant differences in terms of smoking habits (X^2^ = 5.331, *p* = 0.021), insomnia (X^2^ = 4.169, *p* = 0.041) and childhood trauma (X^2^ = 7.636, *p* = 0.006), especially PN (X^2^ = 12.662, *p* < 0.001) between the two groups, while no differences were observed in sex, age, BMI, education level, marriage history, and drinking habits (all *p* > 0.05). In addition, 35 patients experienced at least one type of abuse, including physical abuse (11.4%), sexual abuse (14.3%), emotional abuse (11.4%), physical neglect (85.7%), emotional neglect (40.0%).

**Table 1:** Demographic characteristics of schizophrenic patients with or without suicidal ideation.

	**Without suicidal ideation** **(*N* = 50)**	**With suicidal ideation** **(*N* = 33)**	***t*/X^**2**^**	***p***
			***t***	***p***
Age (year)	40.64 ± 9.32	38.39 ± 10.23	1.033	0.304
BMI (kg/m^2^)	23.72 ± 4.76	24.60 ± 4.32	−0.849	0.398
Total disease course (year)	14.76 ± 8.17	14.27 ± 7.59	0.273	0.785
Age of onset (year)	25.88 ± 9.87	24.12 ± 10.10	0.787	0.433
			**X** ^**2**^	***p***
Sex (male/female)	27 (54%)/23 (46%)	18 (54%)/15 (45%)	0.002	0.961
Education (above grade 6/grade 6 or less)	33 (66%)/17 (34%)	26 (78%)/7 (21%)	1.582	0.209
Married (yes/no)	21 (42%)/29 (58%)	23 (70%)/10 (30%)	1.162	0.281
Physical diseases (with/without)	12 (24%)/38 (76%)	4 (12%)/29 (88%)	1.803	0.179
Drinking history (yes/no)	8 (16%)/42 (84%)	9 (27%)/24 (73%)	1.551	0.213
Smoking history (yes/no)	12 (24%)/38 (76%)	16 (48%)/17 (52%)	5.331	0.021^*^
Insomnia (yes/no)	12 (24%)/38 (76%)	15 (45%)/18 (55%)	4.169	0.041^*^
Childhood trauma (yes/no)	15 (30%)/35 (70%)	20 (61%)/13 (39%)	7.636	0.006^**^
Emotional abuse (EA)	1	3	0.907	0.341
Physical abuse (PA)	1	3	2.032	0.154
Sexually abuse (SA)	2	3	0.233	0.629
Emotional neglect (EN)	8	6	0.365	0.546
Physical neglect (PN)	11	19	12.662	<0.001^***^

*BMI, body mass index*.

*Physical diseases include hypertension, diabetes, head injury, epilepsy, cerebrovascular disease and other endocrine diseases. Drinking history and smoking history means having ever drunk alcohol or smoked. EA ≥ 13, PA ≥ 10, SA ≥ 8, EN ≥ 15, PN ≥ 10 were defined as individuals with a history of childhood trauma*.

* *p < 0.05,*

** *p < 0.01,*

*** *p < 0.001. Data were presented in Mean ± SD or N*.

Table 2 shows the results of the *t*-test of clinical characteristics between schizophrenic patients with and without suicidal ideation. No significant differences were observed between patients with and without SI for positive or negative symptoms, nor for any of the other CTQ dimensions (All *p* > 0.05). Also, there were no significant differences in auditory hallucinations, aggressivity, or depressive symptoms between groups (All *p* > 0.05). There were no significant differences in age, sex, or BMI between the groups (all *P* > 0.05). Patients with suicidal ideation had higher scores of ISI (7.39 ± 4.24 vs. 5.38 ± 3.31, *t* = −2.422, *p* = 0.018) and PN (9.67 ± 3.14 vs. 7.80 ± 2.16, *t* = −2.983, *P* = 0.004) compared to individuals without suicidal ideation. These significant differences remained after controlling for sex, education level, smoking history (*F* = 6.634, df = 1, *p* < 0.05; *F* = 4.652, df = 1, *p* < 0.05), and survived after Bonferroni corrected (all *p* < 0.05).

**Table 2:** Comparisons between schizophrenic patients with and without suicidal ideation.

	**Without suicidal ideation** **(*N* = 50)**	**With suicidal ideation** **(*N* = 33)**	***t***	***p***
ISI	5.38 ± 3.31	7.39 ± 4.24	−2.422	0.018^*^
AHRS	15.74 ± 12.21	17.79 ± 12.78	−0.734	0.465
MOAS	2.20 ± 3.42	4.27 ± 5.90	−1.827	0.074
HAMD	18.44 ± 4.80	20.67 ± 8.32	−1.392	0.171
**CTQ**				
EA	6.38 ± 1.94	7.21 ± 2.79	−1.491	0.142
PA	5.62 ± 1.35	6.70 ± 2.93	−1.976	0.055
SA	5.24 ± 1.14	5.42 ± 1.54	−0.627	0.533
EN	10.32 ± 3.83	11.73 ± 5.41	−1.295	0.201
PN	7.80 ± 2.16	9.67 ± 3.14	−2.983	0.004^**^
Total score	35.36 ± 6.76	40.73 ± 10.65	−2.572	0.013^*^
**BSSI**				
**Suicidal ideation**				
The last week	1.00 ± 0.28	1.29 ± 0.52	−3.182	0.003^*^
The most depressed time	1.07 ± 0.14	1.61 ± 0.61	−5.055	<0.001^***^
**Suicidal risk**				
The last week	0	12.12 ± 20.27	−3.418	0.001^**^
The most depressed time	0	21.12 ± 30.25	−4.011	<0.001^***^
**PANSS**				
Positive subscore	23.60 ± 5.91	23.58 ± 5.39	0.019	0.985
Negative subscore	28.14 ± 4.64	28.33 ± 4.28	−0.194	0.849
General psychopathology	53.34 ± 7.83	52.85 ± 9.14	0.262	0.794
Total score	105.08 ± 12.91	104.91 ± 14.33	0.056	0.955

*ISI, Insomnia Severity Index; AHRS, Auditory Hallucinations Rating Scale; MOAS, Modified Overt Aggression Scale; HAMD, Hamilton Rating Scale for Depression; CTQ, Childhood Trauma Scale; BSSI, The Beck Scale for Suicide Ideation; PANSS, The Positive and Negative Syndrome Scale; EA, Emotional abuse; PA, Physical abuse; SA, Sexually abuse; EN, Emotional neglect; PN, Physical neglect*.

* *p < 0.05*,

** *p < 0.01,*

*** *p < 0.001. Data were presented in Mean ± SD*.

The binary logistic regression analysis found that PN was negatively associated with SI in patients (OR = 5.46, 95% CI = 0.007–0.483, *P* < 0.05) (see Table 3). The adjusted R-square of the whole model was 0.193. Further, a stepwise multiple linear regression was performed, and the results revealed that SI severity was significantly associated with ISI scores (β = 0.272, *P* = 0.011) and PN scores (β = 0.257, *P* = 0.017), with adjusted R-square as 0.177 in this model (see Table 4).

**Table 3:** Results of the stepwise logistic analysis: independent risk factors for schizophrenic patients with suicidal ideation.

**Predictors**	**B**	**SE**	**Wald**	**df**	**Sig**	**Exp.(B)**	**95% CI for Exp.(B)**
Physical neglect	−1.696	0.493	11.820	1	0.001^**^	0.183	0.007–0.483

** *p < 0.01*.

**Table 4:** Results of the stepwise multiple linear regression: significant influencing factors for the severity of suicidal ideation in schizophrenic patients.

**Factor**	**Unstandardized coefficients**		**Standardized coefficients**	**t**	**p**	**95% CI for Exp(B)**	
	**B**	**SE**	**B**			**Lower**	**Upper**
ISI	0.025	0.010	0.272	2.589	0.011^*^	0.006	0.045
PN	0.033	0.014	0.257	2.449	0.017^*^	0.006	0.060

*ISI, Insomnia Severity Index; PN, Physical neglect*.

* *p < 0.05*.

**Figure 1:**
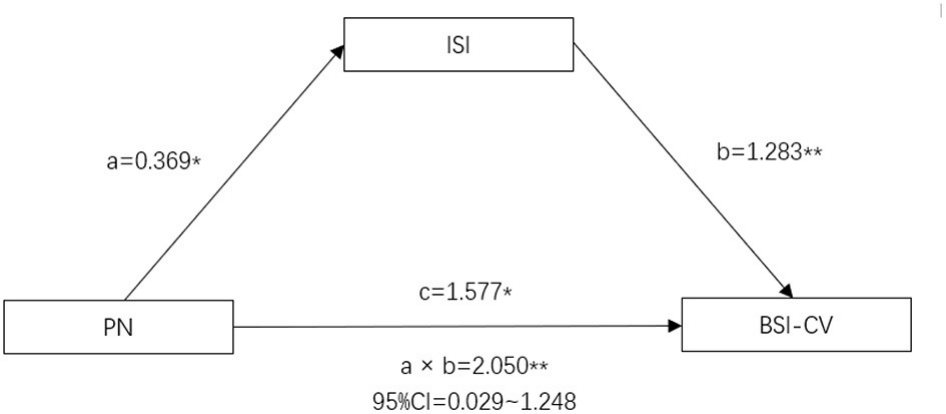
Intermediate model of PN, insomnia and suicidal ideation in schizophrenia patients (*p < 0.05; **p < 0.01). Path a is an independent variable (X) → mediator (M). Path b is mediator (M) → suicidal ideation (Y), adjusted for X. Path c is X → suicidal ideation (Y), adjusted for M. *p < 0.05. **p < 0.01. PN, Physical Neglect; ISI, Insomnia Severity Index; BSI-CV, The Beck Scale for Suicide Ideation.

According to the principle of mediation effect (38), both PN and ISI scores were significantly related to suicidal ideation in the schizophrenia group. Therefore, a mediation model of PN-ISI-suicidal ideation might exist. Based on the theoretical assumptions (39), constructing a mediating effect model of insomnia between PN and suicide, taking the suicidal ideation as dependent variables, using PN score as an independent variable, and using ISI score as an intermediary variable. It was found that the direct effect of PN on suicidal ideation was 1.577 (*p* = 0.003, 95% CI: 0.565–2.589) and the mediating effect of insomnia between PN and suicidal ideation was 1.283 (95% CI: 0.558–2.008). Besides, the total effect of the PN-ISI-suicidal ideation model was 2.050 (95% CI: 1.008–3.093). The interval of indirect effect didn't contain 0, which was statistically significant. Therefore, insomnia played a mediating role between PN and suicidal ideation in schizophrenia patients (Figure 1).

## Discussion

Here, we investigated the association between suicidal ideation, CT, and clinical variables. The study aimed to examine potential relationships between suicidal ideation and different dimensions of CT in patients with schizophrenia to identify a promising way to effectively prevent and ultimately reduce the incidence of suicide.

In this study, we found that schizophrenia with suicidal ideation was related to insomnia and childhood traumatic experiences. Further, binary logistic regression analysis suggested that physical neglect was an independent risk factor for suicide ideation in patients with schizophrenia. According to the result of this study, insomnia truly plays a medicating role in the PN and suicidal ideation. This indicates that PN and insomnia together regulate suicidal ideation in schizophrenia patients. To the best of our knowledge, CT experience may be a promising factor for evaluating the risk of suicide ideation in patients with schizophrenia. Li et al. reported that patients with a history of suicidal behaviors had significantly higher trauma scores than patients without such behaviors (40). Other studies also reported the relationship between CT and suicide. Roy found CT increases the risk for attempting suicide (41). In the present study, patients with CT had higher suicide ideation than those without CT, which is in accordance with previous studies, to a certain extent. CT may increase suicidality in schizophrenia through several biological or psychological mechanisms (13); this question warrants further investigation. The clinical implications of these findings involve the need to prevent suicidality in patients with schizophrenia and the accompanying suggestion that clinicians carefully assess patients' trauma experiences in childhood to provide therapeutic interventions to reduce them.

On the subdomain level, logistic regression analyses indicated that among CT dimensions, only physical neglect was a predictor of current suicidal ideation, but no difference in the exposure to sexual, emotional, or physical abuse. This shows the importance of evaluating and considering the effect of each type of trauma on its own. It has been found that PN is a stronger risk factor for suicide in patients with schizophrenia, compared to other types of trauma such as emotional abuse or neglect (21), which can damage their social function, then worse coping strategies related to negative consequences such as suicidal behaviors (42, 43). This variability of the association between several types of CT and suicidal risk was also found in a prospective study (44). PN of child maltreatment should be considered important risks to suicide. The awareness of the serious long-term consequences of PN should encourage better identification of those at risk and the development of effective interventions to protect children from violence.

The other main findings in our study are that insomnia predicted suicidal ideation independently from any type of CT. Our findings are in line with the growing literature suggesting a significant association of sleep disturbances with suicidal behaviors. A retrospective cohort study showed that insomnia increases the risk of suicide in patients with mental disorders (45). An 8-year longitudinal study of schizophrenia-spectrum disorders also confirmed this hypothesis (46). While those findings support the association between insomnia symptoms and suicide-related risk, the specific mechanisms by which insomnia symptoms confer risk for suicide are still unknown. Several psychological and physiological mechanisms have been proposed (47, 48). Specifically, they reported that negative cognitive appraisals, perceived social isolation, and unhelpful emotion regulation strategies may be potential mechanisms. It is crucial to take the development of targeted psychological interventions for those who have sleep problems.

Moreover, schizophrenia patients have presented correlations among PN, ISI, and suicidal ideation. The PN-ISI-suicidal ideation intermediate model has proved that PN and insomnia can affect suicidality together in schizophrenia patients. Findings from this study have specifically suggested a potential role of the PN-ISI-suicidal ideation loop in the pathogenesis of schizophrenia. Since no study to data explored this loop in schizophrenia patients, more further studies are warranted to verify our preliminary findings.

The current study also has several limitations. First of all, the Childhood Trauma Scale is a subjective questionnaire, which is a retrospective study, resulting in biased reporting. Second, the small sample size not only represents the difficulties in conduction research in this patient group but also affects the robustness of the statistical analyses with a risk of type-II errors which calls for future replication in larger subject samples. And the sample size of specific subdomains in CT was too small to demonstrate weaker associations between two variables. It is possible patients that physical neglect is more likely to report PN over other abuses, which would bias results. One study showed that (PN) and emotional neglect (EN) were most reported, and sexual abuse (SA) and physical abuse (PA) were the least reported (21). Therefore, the results should be taken as preliminary data. Further investigation with a large study population is required to identify the subdomains in CT that might have been overlooked due to poor statistical power and confirm the possible associations that were observed in the present study. Thirdly, the study was cross-sectional. No temporal relationships could be established. Finally, the lack of a normal or clinical control group was a further limiting factor to clarify whether these results are specific to schizophrenia.

In conclusion, schizophrenia with SI reported higher exposure to CT than those without SI. The results point toward childhood physical neglect to be of specific importance to schizophrenia, which may be an area for future prevention and clinical attention. At the same time, our study put forward the intermediate model of PN-insomnia-suicidal ideation while demonstrating insomnia as a full intermediary between PN and suicidal ideation in schizophrenia patients. It may be, then, that addressing adverse childhood experiences and insomnia in these patients at higher risk for suicide because of having suffered CT and insomnia could result in reducing suicides.

## Data Availability Statement

The raw data supporting the conclusions of this article will be made available by the authors, without undue reservation.

## Ethics Statement

The studies involving human participants were reviewed and approved by the Medical Ethics Committee of the Affiliated Kangning Hospital of Wenzhou Medical University. The patients/participants provided their written informed consent to participate in this study.

## Author Contributions

YC, WT, and WX conceptualized and designed the study. YZ, XF, and WT recruited the participants and completed the screening assessments. BT, KF, YC, and NW analyzed the data and performed the statistical analysis. YZ, XF, and KZ wrote the first draft of the manuscript. All authors revised the manuscript and approved the final manuscript.

## Funding

This work was supported by the Science and Technology Program of Wenzhou (Y20190478).

## Conflict of Interest

The authors declare that the research was conducted in the absence of any commercial or financial relationships that could be construed as a potential conflict of interest.

## Publisher's Note

All claims expressed in this article are solely those of the authors and do not necessarily represent those of their affiliated organizations, or those of the publisher, the editors and the reviewers. Any product that may be evaluated in this article, or claim that may be made by its manufacturer, is not guaranteed or endorsed by the publisher.
